# 

**Published:** 1883-04

**Authors:** 


### *South Carolina State Dental Association*

The Thirteenth Annual Meeting of the South Carolina State Dental Association will be held at Aiken, South Carolina on the 17th day of April. 1883.

Dr. C. C. Patrick, President, Charleston. Dr. S. G. Thomson, 1st Vice-President, Abbeville. Dr. W. P. O'Neill 2d Vice-President, Charleston. Dr. A. P. Johnston, Cor. Secretary, Anderson. Dr. R. Atmar Smith, Treasurer, Charleston. Dr. G. F. S. Wright, Recording Secretary, Columbia.

*Voluntary Essays.*—Dr. J. B. Patrick, "Interstitial Growth." Dr. W. P. O'Neill, "Effects of Syphillis on the Teeth." Dr. B. H. Teague, "Cheap Dentistry." Dr. J. B. Patrick, Jr., "Dental Progress.

*The Executive Committee.*—Dr. B. H. Teague, Chairman. Aiken, South Carolina, Dr. J. Rryerson Smith and Dr. W. C. Wilbur, will arrange with Railroads and Hotels.

A written report is expected from the following Committees:

*Operative Dentistry.*—Dr. A. P. Johnstone, Chairman: Drs. J. T. Calvert and E. C. Jones.

*Mechanical Dentistry.*—Dr. R. Atmar Smith, Chairman; Drs. B. J. Quatlebaum and R. C. Roberts.

*Dental Hygiene*—Dr. H. D. Wilson, Chairman: Drs. A. C. Spain, and J. R. Thompson.

*Dental Education.*—Dr. L. Dotterer, Chairman; Drs. G. B. White, and S. G. Thomson.

Two beneficiary Students of Dentistry, on application to this Association, are recommended each year. Your attention is called to an act of the General Assembly, approved February, 1875, entitled "An Act to regulate the practice of Dentistry and protect the people against Empiricism in South Carolina."

At the above time and place, one State Board of Dental Examiners will be in session, viz: Dr. W. S. Brown, of Charleston, President of the Board : Dr. J. B. Patrick, Dr. H. D. Wilson, Dr. G. B. White and Dr. G. F. S, Wright.

*Secretary of the Board.*

---

### *The Texas Dental Association.*

The annual meeting of the Texas Dental Association will be held in Dallas, commencing the first Tuesday in May, 1883, and continuing for three days. All Dentists in good standing are cordially invited to meet with us.

*Officers.*—W. R. CLIFTON, President, Waco. J. L. FOUNTAIN, Vice-President, Bryan. J. H. GRANT, Corresponding Secretary, Austin. J. B. CHESS, Secretary and Treasurer, San Antonio.

*Executive Committee.*—WM. STILES Austin. W. S. CARRUTHERS, Galveston. G. S. STAPLES, Sherman.

---

### *Dental Society of the State of New York.*

Members of the profession who purpose appearing before the Board of Censors for examination for the certificate of this Society, should immediately communicate their intention to Dr. Frank French, Secretary of the Board, at Rochester, and report to him personally the morning of May 8th, 1883, at the Delevan House, Albany. The examinations will begin at 10 A. M. and continue throughout the day or until the list is exhausted. No examinations will be held during the session of the Society.

J. EDW. LINE,
*Secretary.*

### Index to Volume XVI.--Third Series.

Annual Commencement of the Dental Department of the University of Maryland, - - 570
Alveolar Abscess, How to Treat, - - - 29
Abrasion of the Teeth, - - - - - 35
An Investigation into the Effects of Organisms upon the Teeth, etc., - - - - - 1
Association, Southern Dental, - 44, 88, 91, 134, 145
Association, Texas Dental, - - - - 83
Association, Pennsylvania Dental, - - 40, 86, 133
Association, Pittsburgh Dental, - - - 87
Association, New York Dental, - - - 135
Association, National Dental, - - - 88, 134
Alabama State Dental Association, - - - 471
American Dental Association, - 87, 134, 193, 260
Anatomical Anomalies, - - - - - 45
A New Cure for Alveolar Abscess, - - - 47
Alveolar Abscess, - - - - - - 49
A New Fungus, - - - - - - 95
A Triumph of Dentistry, - - - - - 96
American Medical Association, - - - 131, 162
American Dyspepsia, - - - - - 235
A Marvel of Surgery, - - - - - 239
Asphyxiated by a Tooth, - - - - 278
Auspicious Opening of the First Regular Session of of the Dental Department of the University of Maryland, - - - - - 282
Answers to Questions on the Teeth of the Different Races, 322
Acute Conjunctivitis Caused by the Electric Light, - 335
Atkinson, W. B., M. D., - - - - - - 354
A Generous Contribution to the Museum of the Dental Department of the University of Maryland, - 425
A Class of Pulpless Teeth and Their Treatment, - 409
A New Remedy for Spasms, - - - - 430
A Dentist's Wife's Death, - - - - - 576

A Case of Interest, - - - - - 184

American Academy of Dental Science, - - 332

A New Discovery, - - - - - - 477

Annual Commencement of the Dental Department of the University of Maryland, - - 477

Attachment of Artificial Crowns to Natural Roots, - 481

A Case of Gangrena Oris. - - - - - 519

An Idea on Amalgams, - - - - - 526

Are Dentists' Exempt from Jury Duty, - - 529

A New Hypnotic Depresso-Motor, - - - 528

A Useful Invention, - - - - 572

BIBLIOGRAPHICAL.

Manuel of Dental Surgery and Pathology, Coleman and Stelluman, - - - - - 92

Manuel of Dental Anatomy, Charles Tomes, - 93

Dental Metallurgy, Essig, - - - - 375

Coleman's Dental Surgery and Pathology, - 374

Quiz Compounds, No. 1 Anatomy, Potter, - 375

Physicians Visiting List for 1883, - - - 375

The Independent Practitioner, - - 376

Taft's Operative Dentistry, Fourth Edition, - 427

Elements of Dental Materia Medica and Therapeutics, Stocken, - - - - 428

The Pharmacopœia of the United States of America, 428

Barrett, W. C., M. D., D. D. S., - - 155, 337, 494

Blanches, Dr. M., - - - - 124, 180

Bailey, J. J., L. D. S., - - - - - 211

Backache, Its Causes, - - - - - 240

Berry, Dr. A., - - - - - 372

Boyland. G. H., M. D., - - - - 241

Benign Tumors, - - - - - 241

Beane, L. D., M. D., - - - - - 254

Brophy, Truman W., M. D., D. D. S., - - 395

Bromide of Ethyl for Short, Painful Surgical Operations, 433

Buttner, H. F., D. D. S., - - - - 481

Carbolic Acid Poisoning, - - - - - 527

Characteristics of Saliva in Syphilitics, - - - 8

Carpenter, Dr. L. D., - - - - - - 29

Communicability of Syphilis of Suckling, - - 47

Chlorated Tincture of Iodine, - - - 48

Carbolic Acid, Death from, - - - - 47

College Examinations for the Degree of Doctorate, - 96

Connection of Dental with Medical Education, - 135

Congenital Teeth, - - - - - 144

Case of Interest, - - - - - 184

Cancer of Tongue, Removal of Entire Organ, - 186

Combination of Tin and Gold Fillings, - - 224

Color Relation of Metals, - - - - 232

Cause of Death, - - - - - 279

Cement for Rubber, - - - - - 287

Crural Neuralgia Among Dentists, - - - 287

Changes in the Secretion of Milk under Influence of Medicine, - - - - - 380

Caries and Necrosis of the Maxillary Bones, - - 395

Class of Pulpless Teeth, and Treatment, - - 409

Cacciaourrs, Chevalier., M. D., - - - 417

Cotterell, Vinton C., L D. S., - - - - 421

Cells and Their Relation to Organisms, - - 462

Compressed Nitrous Oxide, - - - - 413

Conclusion not Warranted by Facts. - - 574

Coleman, Alfred., L. D. S., - - - - 413

Cohn, Prof. F., - - - - - 452

Chisolm, Prof. Julian J., M. D., - - - 433

Dental Department of the University of Maryland, - - - - 41, 233, 570, 572

Drs. Watt and Taft on First Permanent Molars, - 385

Death from Carbolic Acid, - - - - 47

Dentistry as a Specialty, - - - - 48

Dental Surgeons in the Army and Navy, - - 93

Dunning, Dr. James G., - - - - 113

Dental Education, - - - - - 120

Disorders of Primary Dentition, - - - 124, 180

Dental Society Meetings, - - - - - 155

Decadence of the American Family, - - - 182

Dental Engine for Surgical Use, - - - 192

Diagnosis and Treatment of Benign Tumors, - - 241

Davenport, Dr. E. S., - - - - 268

Dental Education, - - - - - 333

Dental Colleges, - - - - - 372

Dental Caries, Etiology of, - - - - 405

Dental Fistula, - - - - - - 417
Dental Department of the University of California, - 423
Duncan, Dr. O. G., - - - - - 23
Diagram of an Incisor Tooth, - - - - 476
Decolorization of the Hair from Neuralgia, - 480
Diseases of the Dental Pulp, - - - - 557
Dental Society of the State of New York, - - 569
Effects of Organisms upon the Teeth and Jaws, - - 1
Early American Dentistry' - - - - 172
Ether vs. Chloroform, - - - - 176
Erosion : Probable Causes and Treatment, - - 211
Effects of Irritation on Motor Region of the Brain, - 229
Ether, - - - - - - 285
Etiquette, Professional, - - - - 369
Excision of the Tongue, - - - - 384
Etiology of Dental Caries, - - - 405, 446
Excision of Superior Maxillary and Inferior Dental Nerves, 378
Extraction of Teeth in Pregnant Women, - - - 327
Excision of the Tongue, - - - - 480
Ether Spray as a Cure for Neuralgia, - - - 524
Facial Neuralgia, - - - - - 78
Farinaceous Infant Foods, - - - - 130
Faculty Changes, - - - - - 43
Fungus, - - - - - - 95
Follies of Fashion, - - - - - - 128
Fourteenth Annual Meeting of the Southern Dental Association, - - - - 145
Food Adulterations, - - - - - - 237
Fracture of Superior Molar by a Fall, - - 421
French, Frank, D. D. S., - - - - 168
Filling Over-Exposed Nerves, - - - 575
Gangrene and Hemorrhage from a Carious Tooth, - - 191
Generous Contribution, - - - - 425
Gilmour, H. L., D. D. S., - - - - 509
Gold Alloy for Solder, - - - - - 575
Hemorrhage after Teeth Extraction, - - - 431
Histology of Nerves, - - - - - 254, 308
How to Treat Alveolar Abscess, - - - - 29
Harlan, Dr. A. W., - - - - - - 8

Harper, John G., D. D. S., - - - - - 17

Heitzman, Dr. C., - - - - - • - 54

Hemorrhage and Gangrene from a Carious Tooth, - 191

Handpieces for Dental Engines, - - - - 190

Hengst, B. D. A., M. D., - - - - - 519

Investigation into Effects of Organisms of Mouth, - 1

Is this a New Dental Disease ? - - - - 143

Is Tobacco an Antiseptic? - - - - - 168

Illness of Dr. M. H. Webb, - - - - 190

Improvement in Vulcanite, - - - - - 235

Influence of Electric Light on Health, - - 528

King, Dr. L. H., - - - - - - - 35

Kingsley, N. W., D. D. S., - - - - - - 517

Lawson, George., F. R. C. S., - - - - - 419

Lancet in Dentition, - - - - - - 203

Large Odontome Removed from Lower Jaw, - - 46

Life Insurances, - - - - - - 43

Lancing Gums of Children, - - - - - 383

Liability of Dentists, - - - - - - 424

Longnecker, H. C., D. D. S., - - - - - 500

Maryland University Dental Department Building, - 139

Medicinal Value of Vegetables, - - - - 142

Miller, N., D. D. S., - - - - - - -

Microscopic Structure of Malleable Metals, - - 286

Mills, George S., D. D. S., - - 70, 97, 280

Malformation of the Jaws of a Horse, - - 479

Natural Abilities and Mental Requirements in the Study and Practice of Dentistry, - - 500

New York State Dental Association - - - 135

Nervous Force, Its Origin and Physiology, - - 337

Nitrous Oxide, - - - - - - 239, 336

No Salivation from Amalgam Fillings, - - 287

Nitrous Oxide Gas, - - - - - - 523

On the Causes and Treatment ot Tartar - - 23

Origin, Definition and Division of Tissues, - - 54

Operative Dentistry, - - - - - - 70

Oxalate of Lime Calculus, - - - - - 239

Oxy-Phosphate of Zinc, - - - - - 564

Odonto-Chirurgical Society of London, England. - 544

OBITUARY.

Dr. Marshall H. Webb, - - - 426, 474

Dr. William Fiero Edington, - - - - 477

Dr. William H. Allen, - - - - 521

Plaster of Paris vs. Modelling Composition, - - - 515
Pulp Nodules and Their Treatment, - - - - 513
Pericementitis, - - - - - - - 97
Pennsylvania State Dental Association, - 40, 86, 133
Pittsburgh Dental Association, - - - - 87
Physical Diagnosis, - - - - - - 189
Porcelain Crowns, - - - - - - 219
Pulp Treatment, - - - - - - 235
Preparations of Aconite, - - - - 382
Professional Etiquette, - - - - 369
Pierce, George A., M. D., - - - - 462
Professional Courtesy, - - - - 478
Removal of Left Superior Maxilla and Cheek for Sarcoma 419
Removal of Foreign Bodies from the Ear, - - 143
Removal of Naso Pharyngeal Polypus, - - 83
Rights and Liabilities of Dentists at Common Law, - 113
Roddick, Dr., - - - - - -
Rest In Treatment of Heart Disease, - - 236
Review of the Progress in Histology, - - 268
Resolution of Alveolar Abscess into Serous Cyst, - 429
Relaxation for Dentists, - - - - - 494
Seventy-Sixth Annual Commencement of the University of Maryland, - - - - 524
Spence, Dr. Stewart I., - - - - 515
Secondary Dentine, - - - - - - 17
Southern Dental Association, - - 44, 88, 91, 135
Spontaneous? Abrasion of the Teeth, - - 35
Syphilitic Deformity of the Teeth, - - - 188
Section in Oral and Dental Surgery in American Medical Association, - - - 162
Shulze, Dr. W. H., - - - - - - 219
Sozinskey, Thos. S., M. D, Ph. D., - - - 203
Scurvy and Fresh Meat, - - - - - 284
Specialties and Their Advantages, - - - 289
Separation of Silver from Alloys, - - 574
Swallowing Artificial Teeth, - - - - 384
South Carolina State Dental Association, - - 568
To Gild Without a Battery, - - - - - 384
Toothless Persons, - - - - - 383
The Teeth in Diabetes. - - - - 381
Tetanus from a Carious Tooth, - - - - 379

The Use of Tobacco by Boys, - - - - - 283
The Dental Engine Modified for Surgical Use, - 192
Teale, Dr. Pedgin. - - - - - 176
Thompson, A. H., D. D. S., - - - - - 224
Tobacco Smoking, an Experiment, - - 238
The Study of Diseases of Children, - - - 354
The Speed of Thought, - - - - - 376
The Use of Iodiform in Dental Practice, - - 377
Therapeutics and Physiology, - - - 380
Texas State Dental Association, - - - 569
The Amalgam Question, - - - - - 140
Treatment of Diptheria, - - - - - 432
Texas State Dental Association, - - - - 38
Treatment of the Mouth During the Eruption of the Permanent Teeth, - - - - 32
Treatment of Teeth with Dead or Dying Pulps and Alveolar Abscess, - - - - 49
Therapeutic Use of Nitrite of Amyl, - - 94
Townsend, Dr. H. H., - - - - 49
Therapeutic Effect of Oxygen, - - - 96
The New Building of the Dental Department of the University of Maryland, - - - 88
The Baltimore College of Dental Surgery, - - 80
The Connection of Dental and Medical Education, - 135
The Study of the Face as an Index of the Brain, - 504
The Clinics at the Dental Dental Infirmary of the University of Maryland, - - - 522
The Use of Tobacco in Children, - - - 525
Teeth Injured by Tobacco, - - - - 573
To Disguise the Odor of Iodiform, - - 479
The Hygienic Relation of Artificial Dentures, - 542
Uniting Gold to Amalgam in Tooth Filling, - - 417
Valuable Contributions, - - - - 475
Varnish Your Cavities, - - - - - - 383
Vaccine Virus, - - - - - 473
What the Cell can Do, - - . - 452
Warner, Dr. F., - - - - - 504
Withdrawal, - - - - - - 425
Webb, Dr. Marshall H., - - - 426, 474, 500
Watt, George., M. D., D. D. S , - - 405, 446
Webb, Dr. Morrell A., - - - - 500
Zinc vs. Babbitt Metal, - - - - 234

### To Readers and Correspondents.

Communications intended for publication, and exchange journals and papers, should be addressed to the EDITOR OF THE AMERICAN JOURNAL OF DENTAL SCIENCE, 259 N. Eutaw St., Baltimore, Md. All letters relating to the business of the journal, or containing cash remittances, to be directed to Messrs. SNOWDEN & COWMAN. Articles for publication should be received by the editor at least three weeks previous to the first of the month on which the number in which it is designed for them to appear, is issued.

CONTENTS:

ART. I.—Are Dentists' Exempt from Jury Duty? .......... 529

ART. II.—The Hygienic Relations of Artificial Dentures By G A. D. G. .......... 542

ART. III—Odonto-Chirurgical Society of London England .......... 544

ART. IV.—Diseases of the Dental Pulp. By Dr. Samuel A. White .......... 557

ART. V.—Oxy-Phosphate of Zinc. By E. G. Betty, D. D. S. .......... 564

South Carolina State Dental Association .......... 568

Texas State Dental Association .......... 569

Dental Society of the State of New York .......... 569

EDITORIAL, ETC.

University of Maryland, Dental Department, Annual Commencement .......... 5[illegible]0

Alumni Association of the Dental Department of the University of Maryland .......... 572

A Useful Invention .......... 572

MONTHLY SUMMARY.

Teeth Injured by Tobacco .......... 573

Separation of Silver From Alloys .......... 574

A Conclusion not Warranted by Facts .......... 574

Gold Alloy for Solder .......... 575

Filling Over-Exposed Nerves .......... 575

A Dentists' Wife's Death .......... 576

---

☞ The American Journal of Dental Science will contain fifty pages of reading matter in each number, making a volume of about *Six Hundred* pages,

EXCLUSIVE OF ADVERTISEMENTS.

THE

### AMERICAN JOURNAL

OF

### DENTAL SCIENCE.

The *Journal* is issued on the first of each month and contains not less than *fifty* pages of reading matter in each number, exclusive of advertisements, making a volume of *six hundred* pages per annum.

In each number will be found Original Communications, Correspondence, Selected Articles, Monthly Summary, Bibliographical Notices and Editorial Matter, and every effort will be exerted to render the *Journal* worthy of the patronage of the Dental profession.

A generous co-operation is respectfully solicited from the members of the profession; and as the *Journal* has now a printing office of its own, which materially lessens the cost of its publication, it will be issued at the reduced subscription price of

**$2.50 Per Annum in Advance.**

Communications are respectfully solicited on all subjects pertaining to the practice of dentistry, as well as reports of cases occurring in dental practice.

### RATES OF ADVERTISING.

| | 1 MO. | 2 MO. | 3 MO. | 6 MO. | 12 MO. |
|---|---|---|---|---|---|
| 1 PAGE. | $15 00 | $22 50 | $30 00 | $50 00 | $100 00 |
| ½ " | 10 00 | 15 00 | 20 00 | 30 00 | 50 00 |
| ¼ " | 7 00 | 11 00 | 15 00 | 20 00 | 30 00 |
| ⅛ " | 5 00 | 8 00 | 11 00 | 15 00 | 20 00 |

All original communications designed for publication, works for review, new instruments for notice, exchanges, &c., should be directed to the editor, 259 N Eutaw St., Baltimore, Md.

All advertisements and subscriptions should be addressed to

SNOWDEN & COWMAN,

*Publishers of the Am. Journal of Dental Science.*

86 W FAYETTE ST. BET. CHARLES & LIBERTY STS.,

BALTIMORE, MD

